# Hypoxia-Inducible Factor 1α Affects Yak Oocyte Maturation and Early Embryonic Development by Regulating Autophagy

**DOI:** 10.3390/antiox13070840

**Published:** 2024-07-14

**Authors:** Xin Ma, Meng Wang, Jinglei Wang, Xiaohong Han, Xiaoqing Yang, Hui Zhang, Donglan Zhong, Shantong Qiu, Sijiu Yu, Libin Wang, Yangyang Pan

**Affiliations:** 1 College of Veterinary Medicine, Gansu Agricultural University, Lanzhou 730070, China; mxinmaxin@163.com (X.M.); wangmeng@gsau.edu.cn (M.W.); w231012gsau@163.com (J.W.); hanxh1254@126.com (X.H.); 15117062126@163.com (X.Y.); zhui199527@163.com (H.Z.); 17726919564@163.com (D.Z.); qiushantong1998@163.com (S.Q.); sijiuy@126.com (S.Y.); wanglb@gsau.edu.cn (L.W.); 2 Gansu Province Livestock Embryo Engineering Research Center, Lanzhou 730070, China

**Keywords:** yak, oocyte maturation, CYP450s, cumulus diffusion factor, ROS

## Abstract

In animal assisted reproductive technology, the production of high-quality oocytes is crucial. The yak, having lived in the Qinghai-Tibet Plateau for an extended period, has reproductive cells that are regulated by hypoxia-inducible factor 1α (HIF-1α). This study aimed to investigate the impact of HIF-1α on yak oocyte maturation and early embryonic development in vitro through the regulation of autophagy. The in vitro maturation process of yak oocytes involved the addition of the HIF-1α inducer DFOM and the inhibitor LW6 to examine their effects on yak oocyte maturation, early embryonic development, cell autophagy, cytochrome P450s (CYP450s) enzyme expression, and cumulus diffusion factors. The findings revealed that DFOM significantly upregulated the expression of HIF-1α, resulting in increased the cumulus diffusion area, elevated first polar body expulsion rate of oocytes, enhanced mitochondrial and actin levels, decreased ROS production, and reduced early apoptosis levels of oocytes. Moreover, DFOM promoted the expression of autophagy-related proteins, CYP450s enzymes, and cumulus diffusion factors, thereby enhancing oocyte maturation and early embryonic development. Conversely, LW6 exhibited opposite effects. The inhibition of autophagy levels with 3-MA during DFOM treatment yielded similar outcomes. Furthermore, reducing autophagy led to increased apoptosis levels at all stages of early embryonic development, as well as a significant decrease in total cell number and ICM/TE ratio of blastocysts. Studies have shown that during the in vitro maturation of yak oocytes, HIF-1α can affect the cumulus expansion area of oocytes by regulating autophagy, the first polar body excretion rate, mitochondrial level, actin level, ROS and early apoptosis level, the CYP450s enzyme, and the expression of cumulus expansion factors, thereby improving the in vitro maturation and early embryonic development of yak oocytes. These findings offer valuable insights into the reproductive regulation mechanism of yaks in hypoxic environments and suggest potential strategies for the advancement of yak assisted reproductive technology.

## 1. Introduction

The protection of endangered animals is essential for preserving the earth’s biodiversity and ecological equilibrium. Due to increased human activities and the ongoing loss of natural habitats, numerous animal species are at risk of extinction. Safeguarding these species not only aids in maintaining ecosystem stability but also guarantees that future generations can appreciate a diverse natural heritage. The yak, as a high-risk animal, serves as a significant case in endangered species conservation [1]. Yaks, a dominant species adapted to high-altitude environments with strong ultraviolet radiation and low oxygen levels in plateau areas, play a vital role in providing meat, milk, fur, and other essential resources for herdsmen on the Qinghai-Tibet Plateau [2]. Additionally, they serve as a crucial mode of transportation. Despite their importance, yaks have limited reproductive capacity, being seasonal estrus animals from August to November, with a low reproduction rate allowing for reproduction only once every two years [3]. These limitations have hindered the enhancement of yak production performance and pose a significant challenge to the development of the yak industry. Due to climate change and human activities, the yak population has experienced a significant decline and has been listed on the International Union for the Conservation of Nature and Natural Resources (IUCN) Red List of Endangered Species. Over the past three decades, the yak population has decreased by approximately 20–30%. Ecologists warn that without immediate and effective conservation efforts, the yak population could plummet by over 50% in the upcoming centuries [4]. To address this issue and increase income, the implementation of assisted reproductive technology is essential [5]. Notably, the in vitro maturation of oocytes is a key aspect, as it directly impacts the developmental potential of embryos [6]. Due to their high-altitude habitat of 3000–5000 m, yaks exhibit low reproductive rates, as well as limited in vitro oocyte maturation and embryonic development capabilities. These factors contribute to lower pregnancy rates following embryo transfer, increased risk of miscarriage, and reduced live birth rates, highlighting challenges in yak reproduction [7]. Therefore, the development of a high-quality oocyte production strategy is crucial in improving yak fertility [8,9].

Hypoxia-inducible factor 1-alpha (HIF-1α) is a crucial transcription factor that plays a significant role in regulating cellular adaptability to hypoxic environments [10,11]. It is recognized as the primary sensor through which cells respond to hypoxic stress, ultimately promoting cell survival and adaptation in low-oxygen conditions [12]. Yaks, long-term residents of the Qinghai-Tibet Plateau, exhibit unique reproductive characteristics influenced by their specific environmental conditions, impacting gene and protein expression [13]. Research has demonstrated that the development of yak embryos and germ cells in vitro is governed by hypoxia-inducible factors [14,15]. Autophagy, a vital cellular catabolic process involved in component degradation and recycling, is integral to various physiological and biological functions including cell survival, metabolism, and immunity [16,17]. By aiding cells in adapting to stressors like starvation and hypoxia, autophagy plays a role in reducing the risk of diseases and developmental disorders [18]. In mammalian oocyte maturation, autophagy contributes to enhancing mitochondrial distribution, Ca^2+^ content, and cellular resistance to oxidative stress while slowing down apoptosis [19]. Studies have indicated that HIF-1α can trigger autophagy via epigenetic modifications during hypoxia, with the upregulation of HIF-1α inducing autophagy in mouse granulosa cells [20,21].

Members of the cytochrome family, such as cytochrome P450Scc (CYP11A1), cytochrome P450c17 (CYP17A1), and cytochrome P450arom (CYP19A1), play a crucial role in regulating the hypothalamic–pituitary–gonadal axis in animals by catalyzing the cleavage reaction of cholesterol and steroid hormone precursors [22,23,24,25]. Androgens are converted into estrogens in cumulus cells, with ovarian granulosa cells synthesizing androgen to enhance oocyte maturation [26]. Recent studies indicate that HIF-1α can up-regulate estrogen receptors in pulmonary artery endothelial cells, leading to increased steroidogenic enzyme levels in granulosa cells under hypoxia [27,28]. Cumulus expansion, which is essential for oocyte development, involves key factors like hyaluronan synthase 2 (HAS2), prostaglandin-endoperoxide synthase 2 (PTGS2), pentraxin 3 (PTX3), and tumor necrosis factor alpha-induced protein 6 (TNFAIP6) [29]. These factors contribute to cumulus formation and ovulation, with HAS2 synthesizing hyaluronic acid, PTGS2 producing prostaglandins, PTX3 regulating cumulus matrix formation, and TNFAIP6 promoting extracellular matrix synthesis [30,31,32]. Insufficient levels of these factors can result in infertility and impact oocyte fertilization. The luteinizing hormone surge before ovulation stimulates the expression of genes related to hyaluronic acid and prostaglandin production, creating a conducive environment for oocyte development [33].

Currently, there is a lack of comprehensive understanding regarding the role of HIF-1α in the in vitro maturation process of yak oocytes. This study aims to explore the connection between HIF-1α and autophagy, CYP450s, and cumulus diffusion factors in the context of in vitro maturation of yak oocytes. The findings from this research could offer a novel approach to enhancing the quality of in vitro matured yak oocytes, thereby contributing to the advancement of assisted reproduction and breeding biotechnology in yaks.

## 2. Materials and Methods

### 2.1. Chemicals and Reagents

All chemicals and reagents used in this study were obtained from Sigma Chemical (St. Louis, MO, USA). The HIF-1α activity inducer (DFOM) and inhibitor (LW6) were procured from MCE (Monmouth Junction, NJ, USA).

### 2.2. Oocyte Collection and Treatment with DFOM and LW6

Yak ovary samples were collected from a commercial slaughterhouse in Linxia, Gansu Province. Approximately 50 pairs of ovaries were delivered to the laboratory daily over a period of two months. The ovaries were stored in a sterile incubator at 32–36 °C and promptly transported to the laboratory. Follicles with a diameter of 3–8 mm were extracted from the ovarian surface using a 12–18 G sterile syringe needle. Immature cumulus-oocyte complexes (COCs) with intact morphology and at least three layers of granulosa cells were carefully selected under a stereomicroscope. The COCs were washed three times with a preheated egg washing solution (M199 + 5% serum) under a microscope, then transferred to a well-balanced maturation medium (M199 + 10% fetal bovine serum + 100 μg/mL FSH + 50 μg/mL LH + 100 μg/mL penicillin + 100 μg/mL streptomycin). Each 400 µL oocyte maturation medium contains 50 COCs. The oocyte maturation medium was supplemented with HIF-1α activity inducer DFOM (0, 100, 150, 200 μM) and inhibitor LW6 (0, 25, 50, 75 μM) according to the experimental design, with a control group receiving an equal volume of normal saline. After 24 h of culture at 38.5 °C and 5% CO_2_, mature COCs were collected. The oocytes were treated with 0.1% hyaluronidase to remove cumulus cells, rinsed with DPBS, and gently blown with an egg-sucking needle. Oocyte maturation was assessed based on first polar body discharge, and mature oocytes were collected for parthenogenetic-activated embryo production.

### 2.3. Parthenogenetic Activation and Embryo Culture

**Figure 1:**
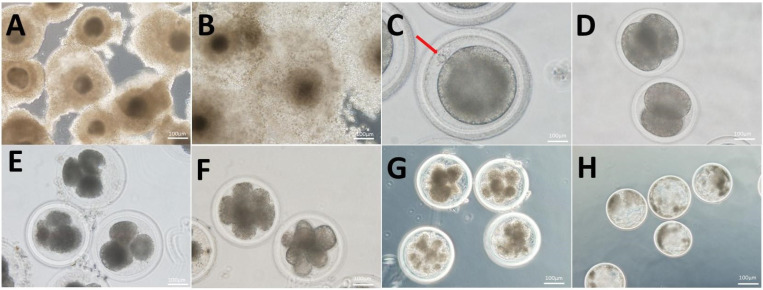
Mature morphology and parthenogenetically activated embryonic development of yak COCs. (**A**) Immature COCs. (**B**) Mature COCs. (**C**) Mature oocytes excrete the first polar body (red arrow). (**D**–**H**) Respectively represent the activation of yak parthenogenetic embryos in the development of 2-cell, 4-cell, 8-cell, morula, and blastocyst stages; bar = 100 μm.

Mature naked eggs were collected and divided into treatment groups with 3 replicates each. Each replicate consisted of 300 naked eggs, with 50 of them being washed 3 times with DPBS and stored at −80 °C for subsequent real-time quantitative polymerase chain reaction. Another 50 eggs underwent DPBS washing and were then placed in immunofluorescence fixative for immunofluorescence staining. A total of 100 eggs were used to assess the developmental potential of yak oocytes. The remaining naked eggs were cultured in modified synthetic oviduct fluid (mSOF, modified synthetic oviduct fluid) after being rinsed three times, exposed to 5 μM ionomycin for 5 min in the dark, and then promptly transferred to fallopian tube fluid medium (mSOF) supplemented with 2 mM 6-dimethylaminopurine (6-DMAP) at 38.5 °C for 4–6 h. The embryos should be washed three times with preheated DPBS for 5 min each time. Subsequently, a group of 45 embryos should be placed in a 35 mm embryo culture dish (174943, Nunclon, Shanghai, China). Within each culture dish, three pre-balanced G1 droplets of culture medium should be placed, with each droplet containing 15 embryos, covered with mineral oil, and cultured under conditions of 38.5 °C, saturated humidity, and 5% CO_2_. The development of yak embryos was monitored at 2-cell (Figure 1D), 4-cell (Figure 1E), 8-cell (Figure 1F), morula (Figure 1G), and blastocyst (Figure 1H) stages at 36, 48, 60, 96, and 192 h, respectively.

### 2.4. Real-Time Quantitative Polymerase Chain Reaction (RT-qPCR)

A set of 50 oocytes per group and total RNA was extracted using the Micro Eute total RNA kit (Omega, Norcross,Georgia, USA). Subsequently, cDNA was synthesized through reverse transcription with the GoScript Reverse Transcription kit (Promega, Madison,Wisconsin, USA). The CDS region of the gene, based on the mRNA sequence of yak stored in GenBank, was selected for RT-qPCR analysis. Primer sequences were designed using Primer Premier 6.0 software and synthesized by Shanghai Shenggong Company. Information for primers used in real-time PCR(Table 1). The reaction mixture consisted of 1.5 μL cDNA, 0.8 μL of forward and reverse primers, 10μL of SYBR Green II fluorescence quantitative PCR Mix (2×), and water to adjust the final volume to 20 μL. This mixture was then subjected to real-time PCR using the Roche 480 instrument (Roche, Basel, Switzerland)under the following conditions: pre-denaturation at 95 °C for 30 s, denaturation at 95 °C for 5 s, annealing at 60 °C for 34 s, and extension at 72 °C for 30 s, for a total of 45 cycles. The internal reference gene, β-actin, was used and the normal saline treatment group served as the control. The mRNA levels in different treatment groups were analyzed using the relative template algorithm and expressed as 2^−ΔΔct^.

### 2.5. Immunofluorescence Staining

The oocytes and embryos from each treatment group (five oocytes in each group) were fixed in 4% paraformaldehyde at room temperature for 1 h, followed by permeabilization with 0.5% TritonX-100 for 45 min. To minimize non-specific antibody binding, we blocked them with 8% BSA for 90 min. Subsequently, the oocytes and embryos were incubated with the primary antibody (the negative control group used PBS instead of antibody working solution) at 4 °C overnight, and then with the corresponding fluorescent secondary antibody at room temperature for 1 h. After washing with DPBS three times, 2.5 ng/mL of DAPI was added and incubated at room temperature in the dark for 3–5 min, followed by three additional washes. Images were captured using the GE DeltaVision Elite living cell workstation. Negative control pictures in supplementary materials (Figures S1–S3).

### 2.6. Detection of ROS Level in Oocytes

After washing the oocytes with 0.5% PVP-PBS three times, they were transferred to DCFH-DA (1:5000, S80033, Beyotime, shanghai, China) and incubated in a CO_2_ cell incubator for 20 min. Subsequently, images were captured using a fluorescence-inverted microscope.

### 2.7. Detection of Actin Level in Oocytes

Following oocyte maturation, oocytes were fixed with 4% paraformaldehyde and stained for actin using the Phalloidin-Fluor TM 594 Conjugate Kit (1:1000, 23122.AAT Bioqucst, Shanghai, China). Subsequently, permeabilization with 0.1% TritonX-100 was carried out for 30 min, followed by transfer to phalloidin droplets, staining at room temperature for another 30 min, and imaging using a fluorescence inverted microscope.

### 2.8. Detection of Mitochondrial Distribution in Oocytes

Mitochondrial distribution in oocytes was visualized using MitoTracker Mitochondrion-Selective Probes (Invitrogen, Carlsbad, CA, USA). The probe, 50 μg, was dissolved in DMSO solution to create a storage solution with a concentration of 1 mM, then diluted at a ratio of 1:5000 and incubated in a cell incubator for 20 min. Images were captured using a fluorescence-inverted microscope.

### 2.9. Detection of Mitochondrial Membrane Potential in Oocytes

After washing the oocytes with 0.5% PVP-PBS three times, they were transferred to JC-1 (1:200, C2006, Beyotime, Shanghai, China) and stained in a CO_2_ cell incubator for 30 min. Subsequently, images were captured using a fluorescence-inverted microscope.

### 2.10. Detection of Early Apoptosis of Oocytes

The level of early apoptosis in oocytes was assessed using the Annexin V-FITC apoptosis detection kit (C1062L, Beyotime, Shanghai, China). A working solution was prepared by mixing 97.5 μL of Annexin V-FITC binding solution with 2.5 μL of Annexin V-FITC. After three washes with 0.5% PVP-PBS, the oocytes were transferred to the Annexin V-FITC working solution and incubated at room temperature in the dark for 25 min. Images were captured using a fluorescence-inverted microscope.

### 2.11. Detection of Apoptosis Level

The level of apoptosis was detected using a TUNEL apoptosis kit (11684795910, Roche, Basel, Switzerland). The samples of different treatment groups were fixed with 4% paraformaldehyde for 45 min and then permeabilized with 0.5% TritonX-100 for 30 min. The 10 μL enzyme solution and 90 μL label solution were mixed to prepare the working solution. After washing with 0.5% PVP-PBS three times, it was transferred to the TUNEL working solution. After 120 min of staining in a CO_2_ cell incubator, the nucleus was stained with DAPI and incubated at room temperature for 5 min. Images were taken using a fluorescence-inverted microscope.

### 2.12. Statistical Analyses

All experimental groups were replicated a minimum of three times. A statistical analysis was conducted using SPSS 25.0 software (Statistical Analysis System Inc., Cary, IL, USA). The comparison between the two groups was assessed using a Student’s *t*-test, while differences among groups were evaluated through one-way ANOVA; Tukey’s test is used as a post hoc test. A *p*-value of less than 0.05 was considered statistically significant. Data are presented as mean ± standard error (mean ± SEM).

## 3. Results

### 3.1. DFOM and LW6 Regulate the Expression of HIF-1α

**Figure 2:**
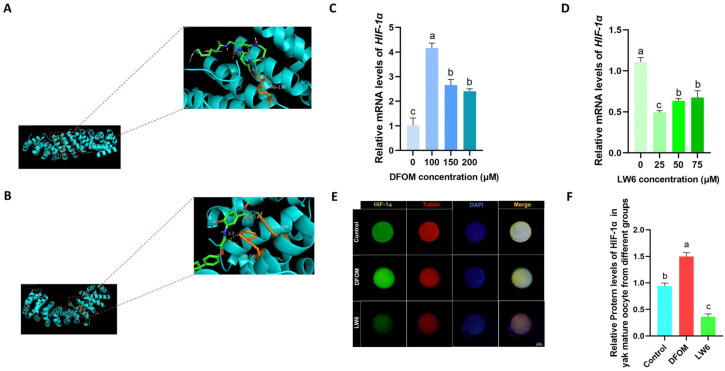
DFOM and LW6 regulate the expression of HIF-1α. (**A**) Molecular docking model of DFOM and HIF-1α. (**B**) Molecular docking model of LW6 and HIF-1α. (**C**) Relative expression level of HIF-1α mRNA after treatment with different concentrations of DFOM. (**D**) Relative expression level of HIF-1α mRNA after treatment with different concentrations of LW6. (**E**) HIF-1α protein in mature oocytes of yak in different treatment groups Immunofluorescence labeling. (**F**) Relative expression levels of HIF-1α protein in mature oocytes of yak in different treatment groups. Bar = 50 μm. Different letters in the histogram indicate statistically significant differences, *p* < 0.05.

To regulate HIF-1α expression in yak oocytes, DFOM, LW6, and HIF-1α were subjected to molecular docking. The findings indicated that DFOM and LW6 are effectively bound to the target with high affinity (Figure 2A,B). Subsequently, different concentrations of HIF-1α inducer DFOM (0, 100, 150, 200 μM) and inhibitor LW6 (0, 25, 50, 75 μM) were introduced into the yak oocyte maturation system. An RT-qPCR analysis revealed that 100 μM DFOM showed the most significant upregulation effect on HIF-1α (*p* < 0.05) (Figure 2C). Similarly, 25 μM LW6 exhibited the most significant down-regulation effect on HIF-1α (*p* < 0.05) (Figure 2D). The combination of 100 μM DFOM and 25 μM LW6 was chosen for the immunofluorescence labeling of the HIF-1α protein in mature yak oocytes (Figure 2E). The results demonstrated that 100 μM DFOM notably upregulated HIF-1α protein levels compared to the control group (*p* < 0.05), while 25 μM LW6 significantly down-regulated HIF-1α protein levels (*p* < 0.05) (Figure 2F).

### 3.2. Effects of HIF-1α on Cumulus Expansion Area and First Polar Body Extrusion Rate of Yak Oocytes

**Figure 3:**
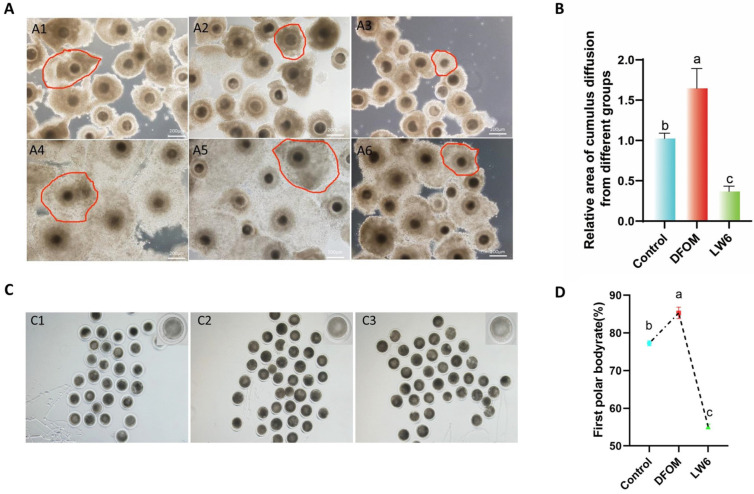
HIF-1α regulates the cumulus expansion area and the first polar body extrusion rate of yak oocytes. (**A**) Cumulus spreading area of oocytes in different treatment groups. (**A1**) Immature COCs in the control group. (**A2**) Immature COCs in DFOM group. (**A3**) Immature COCs in LW6 group. (**A4**) Mature COCs in DFOM group. (**A5**) Immature COCs in the control group. (**A6**) Mature COCs in LW6 group. (**B**) The cumulus expansion area of yak mature oocytes in different treatment groups. **(C)** The first polar body of oocytes in different treatment groups was excreted. (**C1**) The first polar body extrusion of oocytes in the control group. (**C2**) The first polar body extrusion of oocytes in DFOM group. (**C3**) The first polar body extrusion of oocytes in LW6 group. (**D**) The first polar body extrusion rate of yak mature oocytes in different treatment groups. The red frame represents the cumulus expansion area. Bar = 200 μm. The difference of different letters in the histogram was statistically significant (*p* < 0.05).

To determine the cumulus expansion area of yak mature oocytes in different treatment groups, the immature COCs in each group were compared with the mature COCs (Figure 3A). Results indicated a significantly higher cumulus expansion area in the DFOM group compared to the control group (*p* < 0.05), while the LW6 group showed a significant decrease (*p* < 0.05) (Figure 3B). Furthermore, the first polar body excretion rate of each treatment group was assessed. After maturation, cumulus cells were removed with hyaluronic acid to obtain naked eggs, and the first polar body was observed by gently blowing the rotating oocytes with a suction needle under a stereomicroscope (Figure 3C). The results revealed a significantly higher first polar body rate in oocytes from the DFOM group compared to the control group (*p* < 0.05) and a significantly lower rate in the LW6 group compared to the control group (*p* < 0.05) (Figure 3D).

### 3.3. Effect of HIF-1α on the Developmental Potential of Yak Oocytes

**Figure 4:**
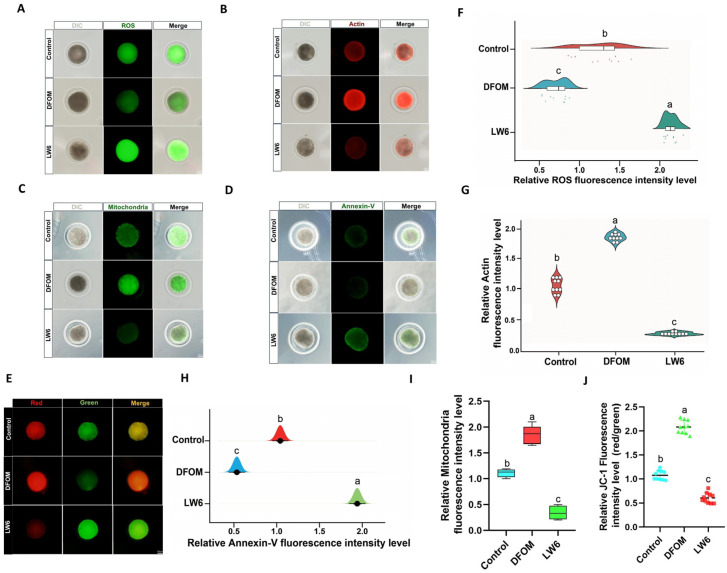
The effect of HIF-1α on the developmental potential of yak oocytes. (**A**) Effects of different treatment groups on ROS in oocytes. (**B**) The effect of different treatment groups on oocyte actin. (**C**) Effects of different treatment groups on the distribution of oocyte mitochondria. (**D**) Effects of different treatment groups on early apoptosis of oocytes. (**E**) The effect of different treatment groups on the distribution of oocyte mitochondria. (**F**) Relative fluorescence intensity of ROS in oocytes of different treatment groups. (**G**) Relative fluorescence intensity of actin in oocytes of different treatment groups. (**H**) Relative fluorescence intensity of oocyte apoptosis in different treatment groups. (**I**) Relative fluorescence intensity of oocyte mitochondria in different treatment groups. (**J**) Effects of different treatment groups on mitochondrial membrane potential of oocytes. Bar = 50 μm. Different letters in the histogram indicate statistically significant differences (*p* < 0.05).

To assess the oxidative stress level of oocytes, DCFH staining was utilized to compare the ROS levels among different treatment groups (Figure 4A). The findings indicated a significantly lower ROS level in oocytes treated with DFOM compared to the control group (*p* < 0.05), while oocytes in the LW6 group exhibited a notably higher ROS level than the control group (*p* < 0.05) (Figure 4F). Actin plays a crucial role in oocyte meiosis, and its levels were assessed using Phalloidin-iFluorTM 488 Conjugate (Figure 4B). The results revealed a significant increase in actin levels when DFOM was added to the oocyte maturation system compared to the control group (*p* < 0.05), whereas LW6 addition led to a significant reduction in actin levels (*p* < 0.05) (Figure 4G). Mitochondria, pivotal organelles responsible for energy generation in cells, greatly influence the developmental potential of oocytes. Mito Tracker^®^ Mitochondrion-Selective Probes were employed to examine the distribution of oocyte mitochondria (Figure 4C). The outcomes demonstrated a significantly higher mitochondrial level in oocytes from the DFOM group compared to the control group (*p* < 0.05), whereas oocytes treated with LW6 exhibited a significantly lower mitochondrial level than the control group (*p* < 0.05) (Figure 4I). Additionally, JC-1 staining was used to assess the mitochondrial membrane potential (ΔΨm) (Figure 4E). The results indicated a significant increase in mitochondrial membrane potential in oocytes from the DFOM group compared to the control group (*p* < 0.05), while oocytes in the LW6 group displayed a significant decrease in mitochondrial membrane potential (*p* < 0.05) (Figure 4J). In addition, the impact of HIF-1α on early apoptosis of oocytes was assessed using the Annexin V-FITC apoptosis detection kit. Results indicated a significant decrease in the Annexin V positive signal in oocytes from the DFOM group compared to the control group (*p* < 0.05). Conversely, oocytes treated with LW6 exhibited a notably higher Annexin V positive signal compared to the control group (*p* < 0.05) (Figure 4D,H).

### 3.4. HIF-1α Regulates the Levels of Autophagy-Related Factors, CYP450s, and Cumulus Diffusion Factors in Yak Oocytes

**Figure 5:**
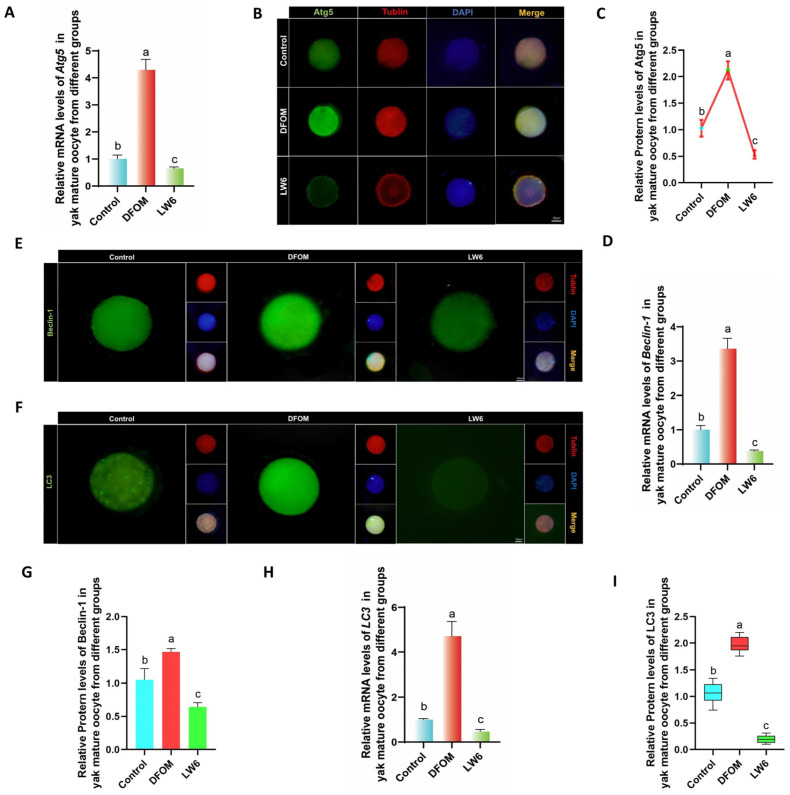
HIF-1α regulates the level of autophagy-related factors in yak oocytes. (**A**,**D**,**H**) The relative expression levels of Atg5, Beclin-1, and LC3 mRNA in oocytes of different treatment groups. (**B**,**E**,**F**) Immunofluorescence labeling of Atg5, Beclin-1, and LC3 proteins in oocytes of different treatment groups. (**C**,**G**,**I**) The relative expression levels of Atg5, Beclin-1, and LC3 proteins in yak mature oocytes of different treatment groups. Bar = 50 μm. The difference of different letters in the histogram was statistically significant (*p* < 0.05).

To investigate the impact of regulating HIF-1α on autophagy levels in oocytes, changes in autophagy-related factors were assessed at the transcriptional and translational levels. An RT-qPCR analysis revealed that Atg5, Beclin-1, and LC3 mRNA levels in oocytes from the DFOM group were significantly higher compared to the control group (*p* < 0.05), whereas the LW6 group exhibited the opposite trend (Figure 5A,D,H). Additionally, immunofluorescence labeling of Atg5, Beclin-1, and LC3 proteins showed that protein levels in oocytes from the DFOM group were significantly elevated compared to the control group (*p* < 0.05), while oocytes from the LW6 group had significantly lower protein levels than the control group (*p* < 0.05) (Figure 5B,C,E–G,I).

**Figure 6:**
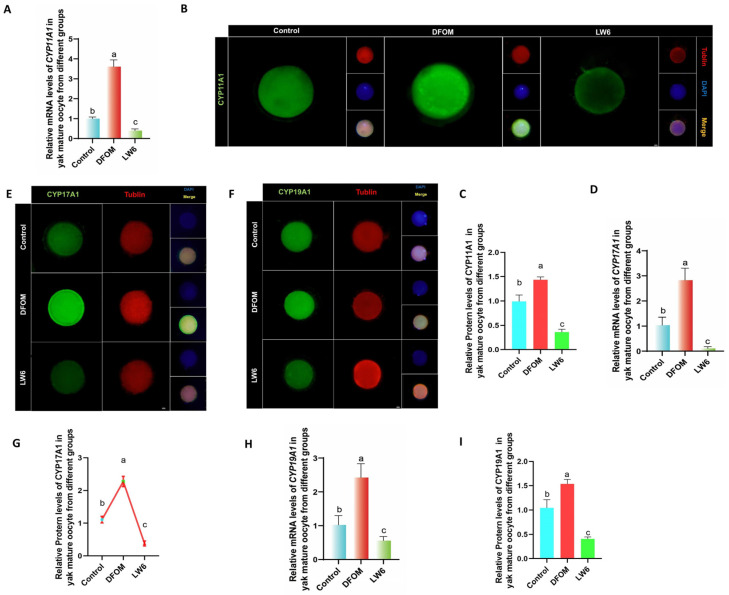
HIF-1α regulates the levels of CYP450s-related factors in yak oocytes. (**A**,**D**,**H**) The relative expression levels of CYP11A1, CYP17A1, and CYP19A1 mRNA in oocytes of different treatment groups. (**B**,**E**,**F**) Immunofluorescence labeling of CYP11A1, CYP17A1, and CYP19A1 proteins in oocytes of different treatment groups. (**C**,**G**,**I**) The relative expression levels of CYP11A1, CYP17A1, and CYP19A1 proteins in yak mature oocytes of different treatment groups. Bar = 50 μm. The difference of different letters in the histogram was statistically significant (*p* < 0.05).

CYP450s were analyzed using RT-qPCR to assess the mRNA levels of CYP11A1, CYP17A1, and CYP19A1 in oocytes from various treatment groups. Results indicated significantly higher mRNA levels of CYP11A1, CYP17A1, and CYP19A1 in oocytes from the DFOM group compared to the control group (*p* < 0.05), whereas the LW6 group exhibited the opposite trend (Figure 6A,D,G). Additionally, immunofluorescence labeling was used to detect CYP11A1, CYP17A1, and CYP19A1 proteins (Figure 6B,E,F). The protein levels of CYP11A1, CYP17A1, and CYP19A1 in oocytes from the DFOM group were significantly higher than those in the control group (*p* < 0.05), while the LW6 group showed significantly lower protein levels (*p* < 0.05) (Figure 6C,G,I).

**Figure 7:**
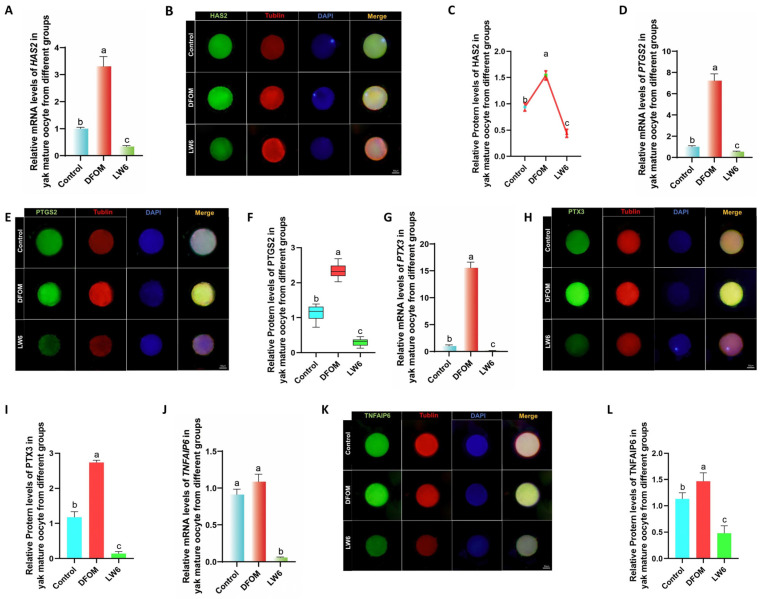
HIF-1α regulates the levels of cumulus diffusion-related factors in yak oocytes. (**A**,**D**,**G**,**J**) Relative expression levels of HAS2, PTGS2, PTX3, and TNFAIP6 mRNA in oocytes in different treatment groups. (**B**,**E**,**H**,**K**) HAS2, PTGS2, PTGS2, immunofluorescence labeling of PTX3 and TNFAIP6 proteins. (**C**,**F**,**I**,**L**) Relative expression levels of HAS2, PTGS2, PTX3, and TNFAIP6 proteins in mature oocytes of yak in different treatment groups. Bar = 50 μm. Different letters in the histogram indicate statistically significant differences (*p* < 0.05).

Changes in cumulus diffusion-related factors in yak oocytes were examined by introducing different treatments to an in vitro culture model. After 24 h, oocytes were retrieved to assess the expression of cumulus diffusion marker genes during in vitro maturation. RT-qPCR analysis revealed that in comparison to the control group, the mRNA levels of HAS2, PTGS2, PTX3, and TNFAIP6 were significantly elevated in the DFOM group (*p* < 0.05), while the LW6 group exhibited the opposite trend (Figure 7A,D,G,J). In order to further verify these results, immunofluorescence labeling was conducted to analyze the expression of HAS2, PTGS2, PTX3, and TNFAIP6 proteins (Figure 7B,E,H,K). The results indicated that the changes in cumulus diffusion-related factors were consistent with mRNA expression trends. Specifically, the protein levels of HAS2, PTGS2, PTX3, and TNFAIP6 were significantly higher in the DFOM group compared to the control group (*p* < 0.05). Conversely, the protein levels of these proteins in the LW6 group were significantly lower than those in the control group (*p* < 0.05) (Figure 7C,F,I,L).

### 3.5. HIF-1α Regulates the Levels of CYP450s and Cumulus Diffusion Factors in Yak Oocytes through Autophagy

**Figure 8:**
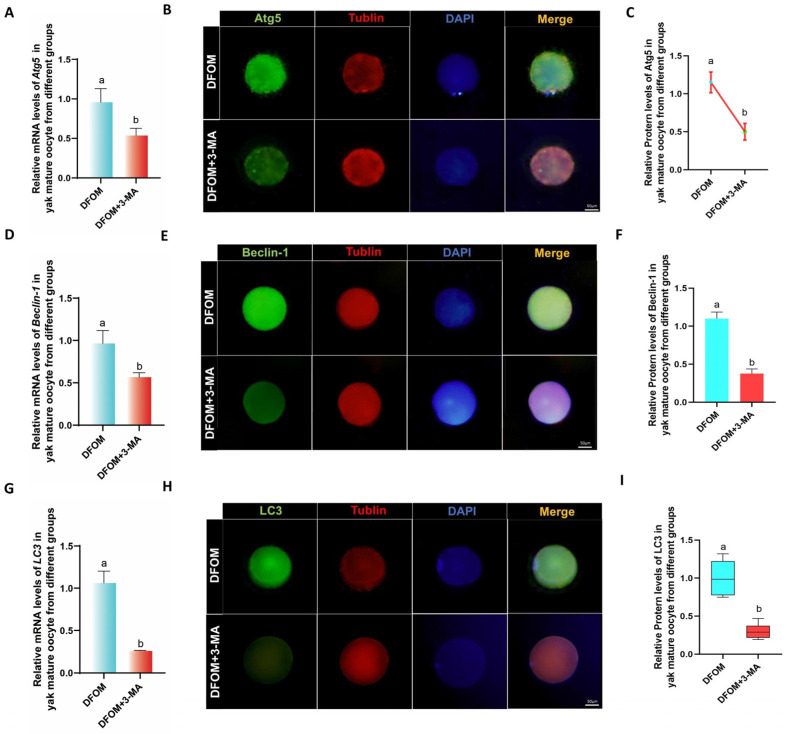
3-MA regulated the level of autophagy-related factors in yak oocytes. (**A**,**D**,**G**) The relative expression levels of Atg5, Beclin-1, and LC3 mRNA in oocytes of different treatment groups. (**B**,**E**,**H**) Immunofluorescence labeling of Atg5, Beclin-1, and LC3 proteins in oocytes of different treatment groups. (**C**,**F**,**I**) The relative expression levels of Atg5, Beclin-1, and LC3 proteins in yak mature oocytes of different treatment groups. Bar = 50 μm. The difference of different letters in the histogram was statistically significant (*p* < 0.05).

In further investigating the potential role of autophagy in the regulation of CYP450s and cumulus diffusion factors, an autophagy-specific inhibitor, 3-MA, was utilized to suppress autophagy levels. A combined treatment group of DFOM and 3-MA was established based on previous findings. The RT-qPCR results revealed a significant decrease in the mRNA levels of Atg5, Beclin-1, and LC3 in the DFOM + 3-MA group compared to the DFOM group (*p* < 0.05) (Figure 8A,D,G). To validate these findings, immunofluorescence labeling of Atg5, Beclin-1, and LC3 proteins was performed (Figure 8B,E,H), showing a consistent trend with mRNA expression (Figure 8C,F,I). These results confirm the efficacy of 3-MA in reducing autophagy levels in oocytes.

**Figure 9:**
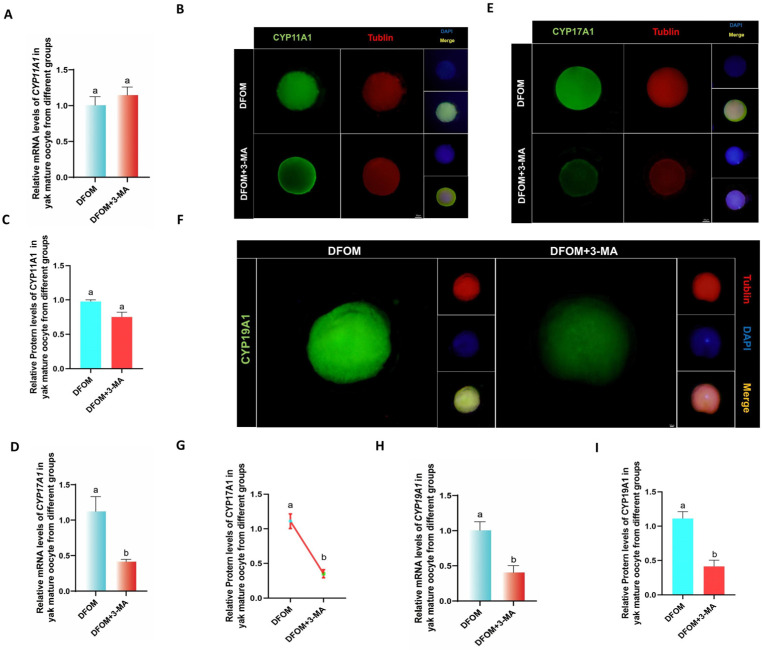
HIF-1α regulates the levels of CYP450s-related factors in yak oocytes through autophagy. (**A**,**D**,**H**) The relative expression levels of CYP11A1, CYP17A1, and CYP19A1 mRNA in oocytes of different treatment groups. (**B**,**E**,**F**) Immunofluorescence labeling of CYP11A1, CYP17A1, and CYP19A1 proteins in oocytes of different treatment groups. (**C**,**G**,**I**) The relative expression levels of CYP11A1, CYP17A1, and CYP19A1 proteins in yak mature oocytes of different treatment groups. Bar = 50 μm. The difference of different letters in the histogram was statistically significant (*p* < 0.05).

RT-qPCR was utilized to assess the levels of CYP450s-related factors CYP11A1, CYP17A1, and CYP19A1 in oocytes following a reduction in autophagy levels. The combined treatment of DFOM and 3-MA resulted in significantly lower levels of CYP17A1 and CYP19A1 mRNA compared to the DFOM group (*p* < 0.05). There was no significant difference in CYP11A1 (*p* > 0.05). (Figure 8A,D,G). The immunofluorescence labeling of CYP11A1, CYP17A1, and CYP19A1 proteins showed a significant decrease in CYP17A1 and CYP19A1 levels in oocytes from the combined treatment group compared to the DFOM group (*p* < 0.05). There was no significant difference in CYP11A1 (*p* > 0.05) (Figure 9B,C,E–G,I). These findings support the role of HIF-1α in regulating CYP450s-related factors in yak oocytes through autophagy.

**Figure 10:**
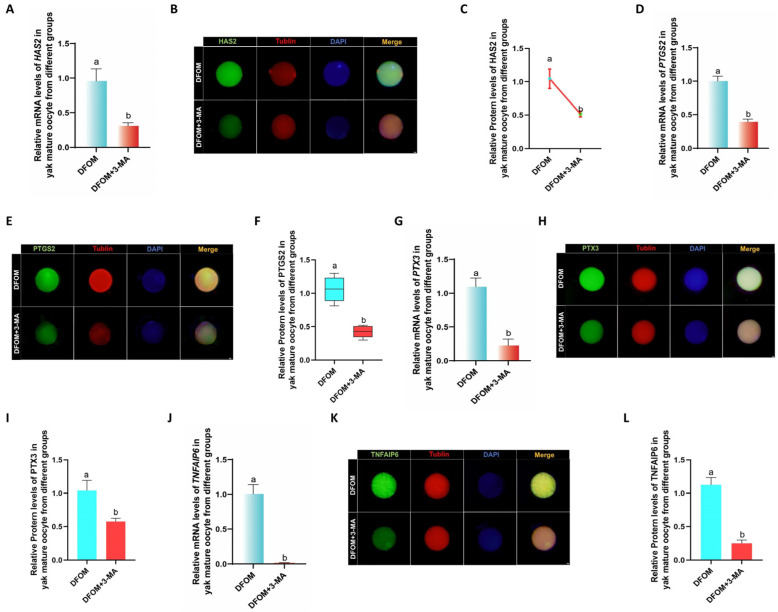
HIF-1α regulates the level of cumulus diffusion-related factors in yak oocytes through autophagy. (**A**,**D**,**G**,**J**) Relative expression levels of HAS2, PTGS2, PTX3, and TNFAIP6 mRNA in oocytes in different treatment groups. (**B**,**E**,**H**,**K**) HAS2, PTGS2, PTGS2, immunofluorescence labeling of PTX3 and TNFAIP6 proteins. (**C**,**F**,**I**,**L**) Relative expression levels of HAS2, PTGS2, PTX3, and TNFAIP6 proteins in mature oocytes of yak in different treatment groups. Bar = 50 μm. Different letters in the histogram indicate statistically significant differences (*p* < 0.05).

To investigate changes in cumulus diffusion-related factors in oocytes following a reduction in autophagy levels, RT-qPCR was utilized to measure the mRNA levels of HAS2, PTGS2, PTX3, and TNFAIP6 in oocytes from various treatment groups. The mRNA levels of these factors in oocytes treated with DFOM combined with 3-MA were significantly lower than those in the DFOM group (*p* < 0.05) (Figure 10A,D,G,J). Subsequently, immunofluorescence labeling of HAS2, PTGS2, PTX3, and TNFAIP6 proteins (Figure 10B,E,H,K) further supported these findings, showing a consistent trend with mRNA expression. Protein levels of these factors in oocytes treated with DFOM combined with 3-MA were also significantly reduced compared to the DFOM group (*p* < 0.05) (Figure 10C,F,I,L). These results confirm that HIF-1α can modulate cumulus diffusion-related factors in yak oocytes through autophagy.

In conclusion, the data demonstrate that HIF-1α plays a role in regulating CYP450s and cumulus diffusion factors in yak oocytes via autophagy.

### 3.6. HIF-1α Regulates the Developmental Potential of Yak Oocytes through Autophagy

**Figure 11:**
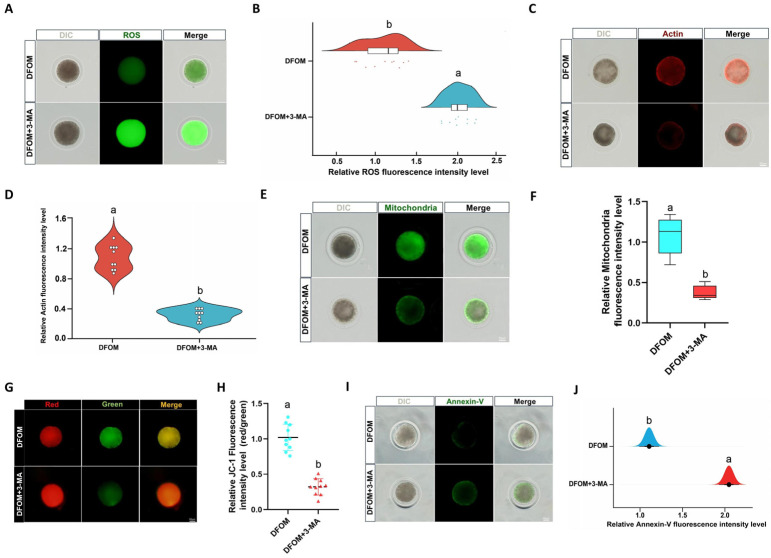
HIF-1α regulates the developmental potential of yak oocytes through autophagy. (**A**) Effects of different treatment groups on ROS in oocytes. (**B**) The relative fluorescence intensity of ROS in oocytes of different treatment groups. (**C**) Effects of different treatment groups on actin in oocytes. (**D**) The relative fluorescence intensity of actin in oocytes of different treatment groups. (**E**) The effects of different treatment groups on the distribution of oocyte mitochondria. (**F**) Relative fluorescence intensity of oocyte mitochondria in different treatment groups. (**G**) Effects of different treatment groups on the mitochondrial membrane potential of oocytes. (**H**) Relative fluorescence intensity of mitochondrial membrane potential in oocytes of different treatment groups. (**I**) Effects of different treatment groups on early apoptosis of oocytes. (**J**) Relative fluorescence intensity of oocyte apoptosis in different treatment groups. Bar = 50 μm. Different letters in the histogram indicate statistically significant differences (*p* < 0.05).

To investigate the impact of reduced autophagy on the developmental potential of oocytes, DCFH staining was utilized to compare ROS levels in different treatment groups (Figure 11A). Results indicated a significant increase in ROS production in oocytes treated with DFOM and 3-MA combined, compared to those treated with DFOM alone (*p* < 0.05) (Figure 11B). Actin levels in oocytes were assessed using Phalloidin-iFluor TM 488 Conjugate staining (Figure 11C), revealing a significant reduction in actin levels with the addition of 3-MA in the oocyte maturation system, as opposed to the DFOM group (*p* < 0.05) (Figure 11D). The mitochondrial distribution was examined using MitoTracker^®^ Mitochondrion-Selective Probes (Figure 11E), showing a lower mitochondrial level in oocytes from the DFOM + 3-MA group compared to the DFOM group (*p* < 0.05) (Figure 11F). The evaluation of mitochondrial membrane potential (ΔΨm) through JC-1 staining (Figure 11G) demonstrated a significant reduction in the DFOM and 3-MA combined treatment group as opposed to the DFOM group (*p* < 0.05) (Figure 11H). Early apoptosis in oocytes was assessed using the Annexin V-FITC apoptosis detection kit (Figure 11I), with results showing a higher level of early apoptosis in oocytes from the DFOM combined with the 3-MA treatment group, compared to the DFOM group (*p* < 0.05) (Figure 11J).

### 3.7. HIF-1α Regulates the Dynamic Expression of Bax/Bcl-2 in Yak Oocytes and Preimplantation Parthenogenetic Embryos through Autophagy

**Figure 12:**
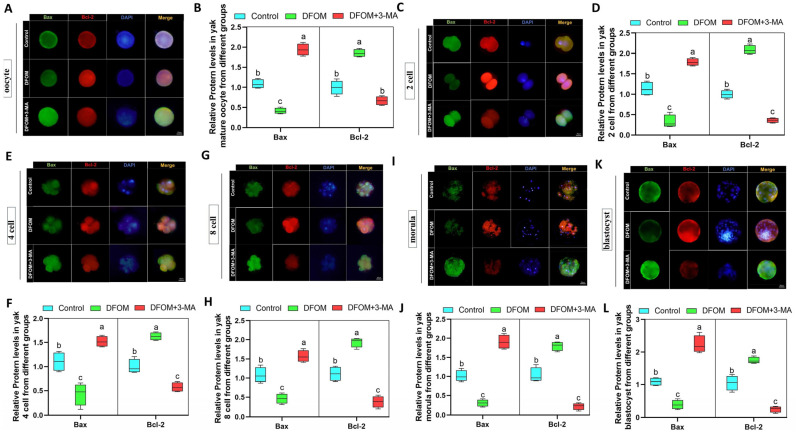
HIF-1α regulates the dynamic expression of Bax/Bcl-2 in yak oocytes and preimplantation parthenogenetic embryos through autophagy. (**A**,**C**,**E**,**G**,**I**,**K**) Immunofluorescence labeling of Bax and Bcl-2 proteins in oocytes, 2 cells, 4 cells, 8 cells, morula, and blastocysts of different treatment groups. (**B**,**D**,**F**,**H**,**J**,**L**) The relative expression levels of Bax and Bcl-2 proteins in oocytes, 2 cells, 4 cells, 8 cells, morula, and blastocysts of different treatment groups. Bar = 50 μm. The difference of different letters in the histogram was statistically significant (*p* < 0.05).

This study investigated the role of HIF-1α in regulating the expression of Bax/Bcl-2 in yak oocytes and preimplantation parthenogenetically activated embryos through autophagy. DFOM was used to modulate HIF-1α levels, while 3-MA was added to decrease autophagy. Immunofluorescence labeling of Bax and Bcl-2 proteins was conducted at different developmental stages of oocytes and embryos (Figure 12A,C,E,G,I,K). The results revealed that the DFOM group exhibited a significant decrease in Bax protein levels and an increase in Bcl-2 protein levels compared to the control group (*p* < 0.05). Conversely, the combination of DFOM and 3-MA treatment resulted in the opposite effect (Figure 12B,D,F,H,J,L).

### 3.8. HIF-1α Regulates Blastocyst Cell Fate and Apoptosis after Parthenogenetic Activation of Yak Oocytes through Autophagy

**Figure 13:**
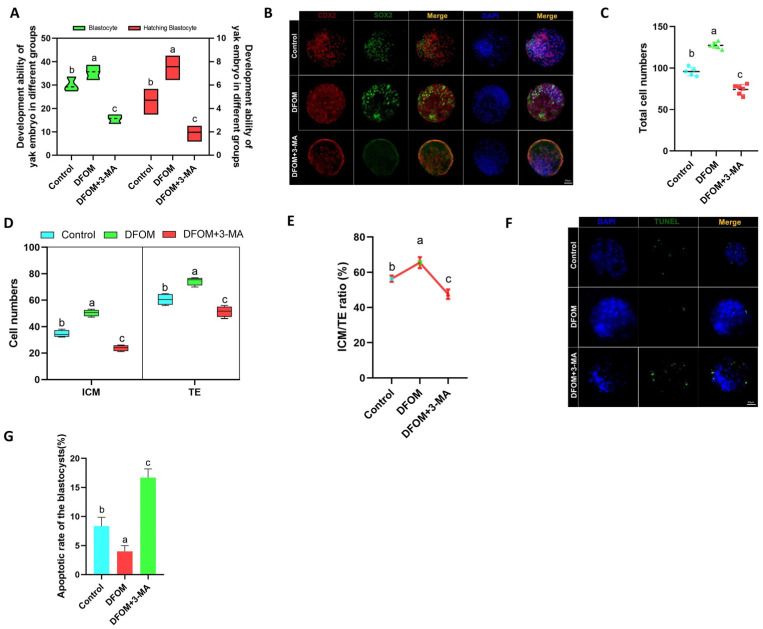
The effect of HIF-1α on the fate and apoptosis of blastocyst cells after parthenogenetic activation of yak oocytes. (**A**) Statistics of blastocyst development rate after parthenogenetic activation of oocytes in different treatment groups. (**B**) Immunofluorescence labeling of CDX2 and SOX2 proteins in blastocysts after parthenogenetic activation of oocytes in different treatment groups. (**C**) Statistics of total cell number of blastocysts after parthenogenetic activation of oocytes in different treatment groups. (**D**) The number of ICM cells and TE cells in blastocysts after parthenogenetic activation of oocytes in different treatment groups was counted. (**E**) The ICM/TE ratio of blastocysts after parthenogenetic activation of oocytes in different treatment groups. (**F**) TUNEL staining of blastocysts after parthenogenetic activation of oocytes in different treatment groups. (**G**) Statistics of blastocyst apoptosis rate after parthenogenetic activation of oocytes in different treatment groups. (Bar = 50 μm). The difference of different letters in the histogram was statistically significant (*p* < 0.05).

To investigate the potential role of HIF-1α in regulating blastocyst development following parthenogenetic activation of yak oocytes through autophagy, the blastocyst rate and hatching blastocyst rate of various treatment groups were analyzed. Results indicated that the blastocyst rate and hatching blastocyst rate of parthenogenetic embryos in the DFOM group were significantly higher compared to the control group (*p* < 0.05), while the DFOM and 3-MA combined treatment group showed the opposite trend (Figure 13A). Subsequent immunofluorescence labeling of ICM and TE of blastocysts using CDX2 and SOX2 proteins revealed an increased total number of blastocysts in the DFOM group, with higher ICM and TE counts. Notably, the ICM/TE ratio was significantly elevated in the DFOM group (*p* < 0.05), suggesting that the upregulation of HIF-1α could enhance blastocyst quality and promote ICM fate, leading to a higher number of ICM cells. The inhibition of autophagy hindered these effects (Figure 13B–E). Furthermore, TUNEL analysis demonstrated that the apoptosis rate of blastocysts in the DFOM group was significantly lower than in the control group (*p* < 0.05), while the DFOM combined with the 3-MA group exhibited a higher apoptosis rate than the control group (*p* < 0.05) (Figure 13F,G).

## 4. Discussion

HIF-1α, a transcription factor activated under hypoxic conditions, is crucial in various biological processes, particularly cell survival and metabolic regulation [34]. Research has demonstrated that HIF-1α enhances cellular adaptability to hypoxia by triggering downstream gene activation [35]. This study specifically investigated the impact of the HIF-1α transcription factor on the in vitro maturation of yak oocytes, suggesting its pivotal role as a mediator connecting environmental stress to oocyte maturation quality.

In this research, DFOM (an inducer of HIF-1α activity) and LW6 (an inhibitor of HIF-1α) were utilized in the treatment of oocytes. This study revealed that DFOM significantly enhanced oocyte maturation rates, whereas LW6 had the opposite effect. Reactive oxygen species (ROS) play a crucial role in oocyte senescence, potentially causing cellular dysfunction if not properly regulated [36]. Our findings demonstrated that the DFOM-induced activation of HIF-1α led to a notable decrease in ROS levels within oocytes, suggesting an enhanced cellular response to oxidative stress. This decrease in ROS levels may be attributed to an improvement in autophagic clearance of damaged mitochondria and other cellular debris, which otherwise could contribute to oxidative stress. Conversely, the inhibition of HIF-1α by LW6 resulted in elevated ROS levels, potentially compromising the integrity and viability of oocytes. This underscores the protective function of HIF-1α in preserving redox balance during oocyte maturation. Vaibhao et al. reported that increasing HIF-1α levels can decrease cellular ROS levels. This finding is in line with the results presented in our research report [37]. The maintenance of mitochondrial integrity and function is crucial for ATP production, oocyte maturation, and embryonic development [38,39]. Our study demonstrated that the activation of HIF-1α improved mitochondrial function by increasing mitochondrial membrane potential, likely through the upregulation of genes involved in mitochondrial biogenesis and the reduction of reactive oxygen species (ROS). This led to a more efficient energy production system in oocytes. Previous research shows that meiosis, a crucial process in oocyte maturation, requires precise cytoskeletal reorganization. HIF-1α may play a role in meiosis by regulating the actin network to ensure proper chromosome distribution during cell division. Treatment with DFOM enhanced actin polymerization, facilitating accurate and efficient meiosis and reducing the risk of chromosomal abnormalities in resulting embryos [40]. Maintaining oocyte quality involves minimizing apoptosis during maturation. Our findings suggest that HIF-1α regulation significantly impacts early apoptosis markers, with DFOM treatment decreasing early apoptosis levels possibly by reducing oxidative stress and enhancing overall cell health. Conversely, LW6 treatment appears to exacerbate apoptosis, underscoring the protective role of HIF-1α in cellular stress. He et al. discovered that HIF-1α in yak has the ability to suppress oocyte apoptosis and enhance oocyte maturation, a finding that aligns with our own research results [9].

Autophagy is essential for maintaining cell health by eliminating damaged organelles and proteins, ensuring a clean intracellular environment vital for normal oocyte maturation [41,42]. Zhang et al. discovered that in H9C2 cells, HIF-1α has the ability to elevate autophagy levels [43]. Our findings demonstrate that activating HIF-1α with DFOM notably boosts the expression of autophagy markers like Atg5, Beclin-1, and LC3, which are crucial for forming autophagosomes. This indicates that increased autophagy flux is a protective response facilitated by HIF-1α under hypoxic stress, which is particularly beneficial for oocytes needing robust mechanisms to combat oxidative stress and ensure proper cytoplasmic maturation. The mechanism involves HIF-1α enhancing the expression of autophagy-related genes, such as LC3 and Beclin-1, which are directly involved in forming autophagic vesicles to eliminate damaged components and maintain intracellular stability. These results suggest that HIF-1α may enhance oocyte maturation rate and quality by promoting autophagy. Previous studies have shown that HIF-1α promotes autophagy in mouse granulosa cells, which is consistent with the findings in this study [21]. This was further confirmed when they were treated with 3-MA, an autophagy inhibitor, which significantly dampened autophagy activity, leading to reduced oocyte maturation rate and developmental potential. This highlights the critical role of autophagy in oocyte maturation, with HIF-1α positively regulating the autophagy process to adapt to hypoxic and other stress conditions.

CYP450s are a group of enzymes essential for the synthesis of steroid hormones, such as CYP11A1, CYP17A1, and CYP19A1. These enzymes play key roles in cholesterol side chain cleavage and sex hormone biosynthesis [25,44,45]. After the DFOM treatment of yak oocytes, there was a significant increase in mRNA and protein expression levels of CYP11A1, CYP17A1, and CYP19A1. This upregulation suggests that HIF-1α activation promotes the expression of steroid hormone synthase in oocytes, potentially enhancing oocyte maturation, fertilization capability, and subsequent development. Conversely, LW6 had the opposite effect. Vijay et al. discovered that HIF1-driven transcriptional activity in bovine granulosa cells plays a role in regulating steroidogenesis and proliferation, aligning with our own research [46]. These findings underscore the regulatory influence of HIF-1α on the reproductive physiology of yaks at high altitudes. Given their unique adaptation to plateau environments, the reproductive system’s adaptive regulation in yaks is crucial for reproductive success. In high-altitude settings, hypoxia is common, and HIF-1α, as a key responder to hypoxia, may regulate CYP450s as a fundamental biological mechanism for yak adaptation [47]. Through the promotion of steroid hormone synthase expression, HIF-1α activation could enhance the synthesis of hormones like estrogen and progesterone in oocytes, thus optimizing the oocyte maturation environment and improving fertilization potential and early embryonic development. This hormonal environment optimization is crucial for enhancing the reproductive efficiency of seasonal estrus yaks. Intriguingly, our research also revealed that decreased autophagy activity was associated with reduced CYP450 levels. Zhang et al. discovered that inhibiting autophagy levels with 3-MA leads to a decrease in steroid hormone synthase in yak granulosa cells, supporting our perspective [48,49].

Cumulus spreading factors, including HAS2, PTGS2, PTX3, and TNFAIP6, are crucial for oocyte maturation and ovulation [50]. This study demonstrates that HIF-1α influences the cumulus structure and function by modulating the expression of these factors, thereby promoting oocyte maturation and ovulation. These findings align with previous research in goats and humans [51,52,53]. The experiment manipulated HIF-1α expression using the inducer DFOM and the inhibitor LW6. Increasing HIF-1α activity significantly enhanced the expression of cumulus diffusion factors. Specifically, DFOM treatment boosted mRNA and protein levels of HAS2, PTGS2, PTX3, and TNFAIP6, leading to cumulus expansion and oocyte maturation. Conversely, LW6 application suppressed the expression of these diffusion factors, potentially impacting oocyte development and ovulation efficiency. The impact of HIF-1α on oocyte development was confirmed through measurements of the cumulus expansion area. DFOM treatment notably increased the cumulus diffusion area and the rate of first polar body extrusion in oocytes, which is indicative of improved mature oocyte quality. Furthermore, co-treatment with DFOM and 3-MA resulted in the decreased developmental potential of oocytes, CYP450s, and cumulus diffusion factors. This decrease in autophagy levels counteracted the upregulation effect of HIF-1α on cells, highlighting the role of HIF-1α in modulating oocyte maturation through autophagy regulation.

HIF-1α plays a crucial role in regulating both autophagy during oocyte maturation and the development of preimplantation embryos. Research has demonstrated that HIF-1α assists embryos in adapting to potential hypoxic conditions by controlling nutrient absorption, energy metabolism, and antioxidant defense mechanisms [54,55]. Additionally, HIF-1α influences embryonic cell apoptosis by modulating the expression of apoptosis-related genes like Bax and Bcl-2. By promoting glycolysis and enhancing energy supply, HIF-1α can impact mitochondrial function and regulate cellular energy levels, which is essential for maintaining oocyte and early embryo viability. The regulation of HIF-1α extends beyond enhancing survival and inhibiting apoptosis during blastocyst development; it also significantly influences the differentiation of inner cell mass (ICM) and trophoblast (TE) cells in the blastocyst stage. Immunofluorescence staining with specific cell markers revealed that DFOM treatment increased the proportion of ICM cells and preserved the health of TE cells, suggesting that the proper activation of HIF-1α can optimize embryo structure and enhance preimplantation development quality and implantation efficiency. He et al. demonstrated that HIF-1α has the ability to improve preimplantation embryo development and influence the cell fate of blastocysts, aligning with our own research results [56]. Similarly, reducing autophagy levels in early embryos led to increased apoptosis at each stage, decreased total blastocyst cell numbers, and a notable decrease in the ICM/TE ratio. These findings underscore the role of HIF-1α in modulating preimplantation embryo development through the autophagy pathway, influencing blastocyst cell fate and apoptosis.

Yaks live in the plateau environment for a long time and are seasonal estrus livestock. Compared to other livestock species, yaks have relatively low reproductive capacity. Enhancing reproductive regulation technology for yaks is a key focus for efficient breeding practices, with a particular emphasis on developing a high-quality oocyte production strategy [57]. While our research highlights the significance of HIF-1α in yak oocyte maturation, it is important to acknowledge the limitations of our study. The primary constraint lies in the in vitro approach, which, despite its informative value, may not completely mirror the intricate in vivo conditions. Subsequent investigations should prioritize translating these results into real-world scenarios, assessing the feasibility and effectiveness of employing HIF-1α modulators in yak breeding initiatives. The findings of this study highlight the potential for optimizing the in vitro maturation of yak oocytes through the regulation of HIF-1α activity to modulate autophagy. This approach holds significant importance in enhancing breeding techniques, improving embryo quality, and increasing the success rate of yak reproduction.

## 5. Conclusions

**Figure 14:**
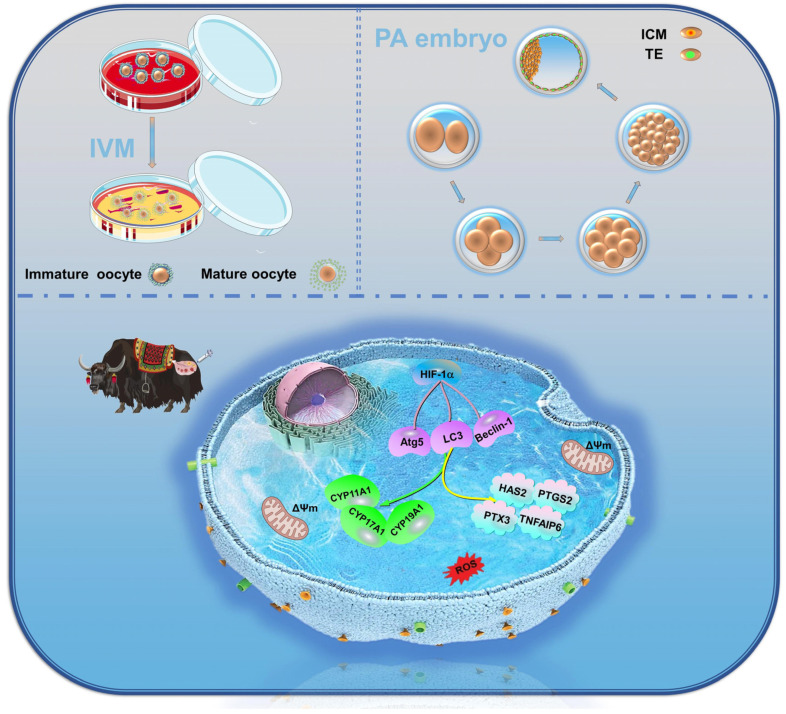
HIF-1α affects yak oocyte maturation and early embryonic development by regulating autophagy.

This study highlights the significant role of HIF-1α in yak reproductive biology, particularly its impact on various aspects of oocyte quality, such as spreading area, first polar body efflux rate, mitochondrial and actin levels, ROS and early apoptosis levels, CYP450s enzymes, and oocyte mound spreading factor expression. These effects are mediated through the regulation of autophagy to improve the in vitro maturation of yak oocytes and early embryonic development (Figure 14). This discovery holds great significance in enhancing our understanding of the reproductive adaptability mechanism of plateau animals and offers new insights and strategies for the future development of yak reproductive technology. Further research is essential to uncover the underlying regulatory mechanisms and implement more effective measures to enhance the reproductive efficiency and success of livestock in alpine regions.

## Figures and Tables

**Table 1:** Information for primers used in real-time PCR.

Gene	Primer Sequences 5′–3′	Tm/°C	Accession Number
*HIF-1α*	F: TGAAGGCACAGATGAATTGCTT R: GTTCAAACTGAGTTAATCCCATGT	56	KU353607.1
*Atg5*	F: AGTTGCTCCTGAAGATGGGG R: TCTGTTGGTTGCGGGATGAT	59	NM_001034579.2
*Beclin-1*	F: GAAACCAGGAGAGACCCAGG R: GTGGACATCATCCTGGCTGG	58	NM_001033627.2
*LC3*	F: CCGACTTATCCGAGAGCAGC R: TGAGCTGTAAGCGCCTTCTT	56	NM_001001169.1
*CYP11A1*	F: CCTCTACTGCCTCCTGAA R: ATCTCGTACAAGTGCCATT	57	NM_176644.2
*CYP17A1*	F: GCCCAAGACCAAGCACTC R: GGAACCCAAACGAAAGGA	60	NM_174304.2
*CYP19A1*	F: GATTTCGCCACTGAGTTGATT R: TCTGGATTTCCCTTATTATTGC	56	NM_174305.1
*HAS2*	F: ACTCATTCCCGTATCCGTTTG R: TTCTTCCGCCTGCCACATT	56	NM_174079.3
*PTGS2*	F: TTCTCGTGAAGCCCTATGA R: GAGGCAGTGTTGATGATTTT	58	NM_174445.2
*PTX3*	F: GCTATCGGTCCATAATGCTTGT R: CTTTCTTTGAATCCCAGGTGC	57	NM_001076259.2
*TNFAIP6*	F: CAAAGGAGTGTGGTGGTGTGTT R: TTCAACATAGTCAGCCAAGCAA	56	NM_001007813.2
*β-Actin*	F: GCGGCATTCACGAAACTA R: TGATCTTCATTGTGCTGGGT	56	DQ838049.1

## Data Availability

All data presented in this study are available on request from the corresponding authors.

## Supplementary Materials

The following supporting information can be downloaded at https://www.mdpi.com/article/10.3390/antiox13070840/s1: Figure S1: Negative control of immunofluorescence target protein in oocytes; Figure S2: Negative control of immunofluorescence target protein in embryo; Figure S3: Negative control of fluorescence staining of oocyte developmental potential.
