# Supplementary material for: Hypoxia-Inducible Factor 1α Affects Yak Oocyte Maturation and Early Embryonic Development by Regulating Autophagy
Source: Antioxidants (Basel). 2024 Jul 14;13(7):840. doi: 10.3390/antiox13070840 (PMC11273763; doi:10.3390/antiox13070840)
Supplement: Supplementary file 1 [file antioxidants-13-00840-s001.zip › antioxidants-3051047-supplementary.pdf]

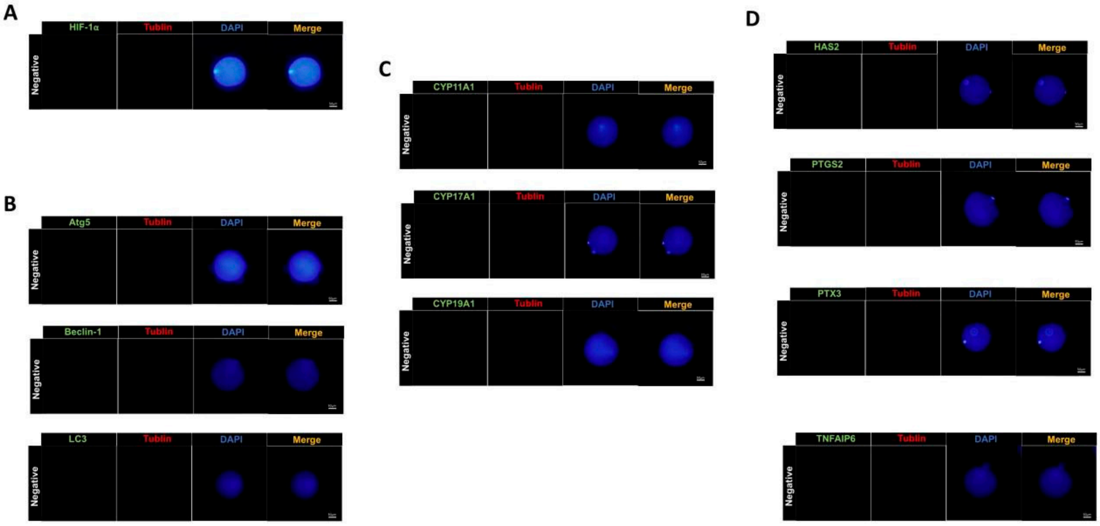

Figure S1. Negative control of immunofluorescence target protein in oocytes. (**A**) Negative control of HIF-1α in oocytes. (**B**) Negative control of autophagy factors in oocytes. (**C**) Negative control of CYP450s in oocytes. **(D)** Negative control of cumulus diffusion factor in oocytes. Bar = 50μm.

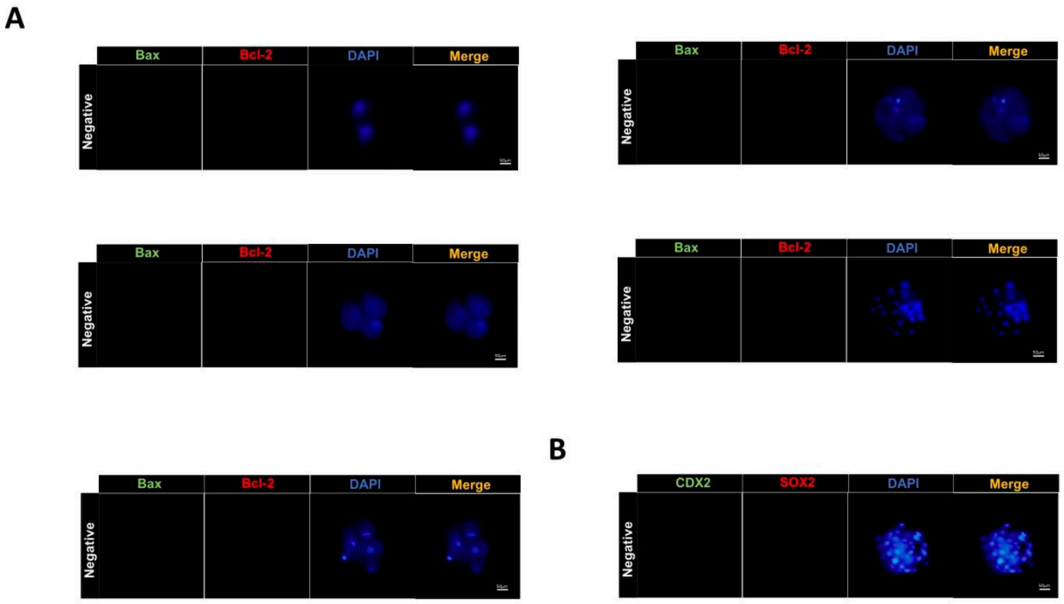

Figure S2. Negative control of immunofluorescence target protein in embryo. (**A**) Negative control of Bax/Bcl-2 in embryo. (**B**) Negative control of CDX2, SOX2 in blastula. Bar = 50μm.

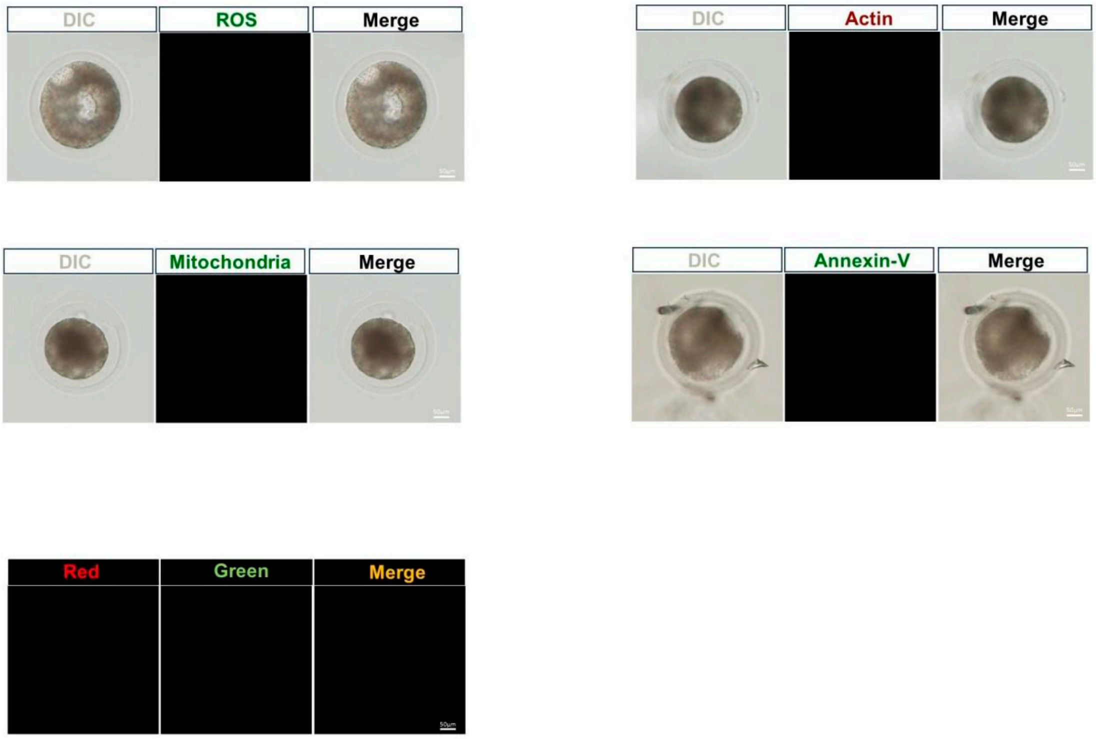

Figure S3. Negative control of fluorescence staining of oocyte developmental potential.
